# Sociodemographic Correlates of Psychotic Experiences in a Non-Clinical Arab Population

**DOI:** 10.1192/j.eurpsy.2026.11317

**Published:** 2026-07-31

**Authors:** L. S. Chaibi, A. Kammoun, W. Cherif, M. Cheour, F. Fekih-Romdhane

**Affiliations:** 1 Faculty of Medecine of Tunis; 2 Psychiatry; 3 Ibn Omran, Razi Hospital, Tunis, Tunisia

## Abstract

**Introduction:**

Psychotic experiences (PEs) are prevalent in the general population, but sociodemographic correlates remain understudied in Arab contexts.

**Objectives:**

This study examines the association between sociodemographic factors and PEs among young adults.

**Methods:**

Data were collected from 7,646 participants across 12 Arab countries using the Prodromal Questionnaire-Brief (PQ-B). Sociodemographic variables included sex, age, marital status, education, and lifestyle habits (smoking, alcohol, cannabis). Bivariate analyses and regression models were applied.

**Results:**

Females had higher PQ-B scores than males (*p* = .043), and single individuals scored higher than married participants (*p* < .001). Lower education levels (secondary or less) and smoking were associated with elevated PEs (*p* = .026 and *p* = .012, respectively). Jordan and Morocco showed the highest PQ-B scores (*p* < .001).

**Conclusions:**

Sex, marital status, education, and smoking are significant correlates of PEs. Culturally tailored mental health strategies should address these risk factors in Arab youth.

**Disclosure of Interest:**

None Declared

